# The association of workplace psychosocial factors on premenstrual dysphoric disorder: A six‐month prospective study on Japanese female workers

**DOI:** 10.1002/pcn5.70313

**Published:** 2026-03-08

**Authors:** Mako Iida, Kazuhiro Watanabe, Miho Egawa, Yuka Ito, Yoshiaki Kanamori, Rikako Tsuji, Daisuke Nishi, Natsu Sasaki

**Affiliations:** ^1^ Department of Mental Health, Graduate School of Medicine The University of Tokyo Tokyo Japan; ^2^ Department of Public Health Kitasato University School of Medicine Kanagawa Japan; ^3^ Department of Gynecology and Obstetrics Kyoto University, Graduate School of Medicine Kyoto Japan; ^4^ Department of Psychiatric Nursing, Graduate School of Medicine The University of Tokyo Tokyo Japan

**Keywords:** female workers, job control, job stability, premenstrual dysphoric disorder, psychological distress

## Abstract

**Aim:**

This study examined the longitudinal associations between psychosocial factors in the workplace and the emergence of premenstrual dysphoric disorder (PMDD) symptoms among Japanese female workers, with a particular focus on psychological distress as a potential moderator.

**Methods:**

We conducted a 6‐month follow‐up of 2000 full‐time Japanese female workers aged 20–39 without PMDD symptoms at baseline. PMDD symptoms were assessed at follow‐up using a validated PMDD scale. Psychosocial factors in the workplace (job demands, job control, supervisor and coworker support, and workplace rewards) were measured at baseline using the New Brief Job Stress Questionnaire. Psychological distress was assessed using the Kessler 6 psychological distress scale (K6), and participants were stratified by K6 scores (K6 < 5 and K6 ≥ 5). Logistic regression analyses were performed.

**Results:**

Of the eligible participants, 1064 completed both surveys (response rate: 61.6%). In the adjusted models, high job control was associated with the emergence of PMDD symptoms among all participants (odds ratio [OR] = 1.26, *p* = 0.025) and those with high psychological distress (OR = 1.37, *p* = 0.011). Job stability was negatively associated with the emergence of PMDD symptoms among participants with high psychological distress (OR = 0.48, *p* = 0.010). No significant associations were found among those with low psychological distress.

**Conclusion:**

Job stability was a protective factor for the emergence of PMDD symptoms among female workers with high psychological distress, while job control may act as an enhancing factor in the overall sample and among those with high psychological distress. Ensuring job stability might be a measure to prevent PMDD symptoms, especially for female workers with high psychological distress.

## INTRODUCTION

Premenstrual symptoms are a prevalent and significant health issue for female workers. Premenstrual syndrome (PMS) and premenstrual dysphoric disorder (PMDD) are conditions characterized by exacerbated menstruation‐related symptoms, either physically or psychologically.^1^ PMDD is characterized by predominantly affective symptoms accompanied by functional impairment. The condition exhibits stricter criteria than PMS and represents the most severe spectrum of premenstrual disorders.^1^, ^2^ According to a meta‐analysis, the point prevalence of PMDD of a community sample is estimated to be 1.6% using confirmed diagnosis, and the pooled prevalence was 3.2% for confirmed diagnosis and 7.7% for provisional diagnosis.^3^ Systematic reviews and meta‐analyses reported that PMDD is associated with increased risks of suicidality^4^, ^5^ and other mood disorders.^6^ Impairment at work is also remarkable. For example, 3%–16% of women were reported to leave their jobs because of PMS.^7^ In addition, PMS and PMDD have been associated with low productivity, such as low presenteeism in the workplace.^8^ Urgent measures are needed to alleviate the effects of these conditions and support female workers with PMDD.

Since the etiology of PMDD involves complex interactions of hormones, genetic factors, and environmental and psychosocial factors,^9^ psychosocial factors in the workplace may also play a critical role.^10^ A systematic review of prospective cohort studies revealed that females with high job demands and low job control were less likely to conceive. Moreover, working over 40 hours per week, engaging in frequent heavy lifting, and rotating night shift work increased the risk of earlier menopause.^11^ Epidemiological data have revealed that female workers in caregiving and physically demanding occupations report higher rates of PMS.^12^ A cross‐sectional study found that low job control, low coworker support, and insufficient job stability were correlated with a higher risk of menstrual pain.^13^ In addition, qualitative studies have suggested the need for more robust workplaces to support female workers with PMDD.^14^ These findings collectively indicate that modifying psychosocial factors in the workplace could contribute to the alleviation of PMDD symptoms.

While existing evidence suggests that psychosocial factors in the workplace may be relevant to menstrual‐related health outcomes, longitudinal evidence regarding the association between job resources and PMDD symptoms is limited. Previous cross‐sectional studies have indicated that female workers with PMS tend to report low job control,^8^ limited coworker support,^15^ and infrequent disclosure of PMS symptoms to supervisors,^8^ that is, inadequate supervisor support. However, these studies focused primarily on PMS or general menstrual symptoms and were unable to clarify temporal relationships or account for workers' mental health status. Drawing on the Demand–Control–Support (DCS) and Effort–Reward Imbalance (ERI) models, insufficient job resources and excessive demands are theorized to exacerbate stress‐related health outcomes, whereas adequate control, social support, and rewards may function as protective factors.^16^, ^17^, ^18^ Emerging evidence further suggests that psychological distress may mediate or modify the relationship between workplace stressors and menstrual‐related symptoms.^15^ Nevertheless, no longitudinal study has simultaneously examined psychosocial job resources, job demands, and mental health status in relation to the emergence of PMDD symptoms.

This study aims to investigate whether psychosocial factors in the workplace—specifically psychosocial job resources (job control, supervisor and coworker support, and workplace rewards) and job demands—are associated with the new emergence of PMDD symptoms 6 months later among female workers in Japan, hypothesizing that job resources are protective, whereas job demands increase risk. To consider the impact of mental health status, participants were stratified into groups with high and low psychological distress, and the relationships were examined.

## MATERIALS AND METHODS

### Study design

This longitudinal study used data from an online survey of full‐time female workers in Japan (*n* = 2000). The baseline survey was conducted in August 2023, and the follow‐up survey was conducted in February 2024. The Graduate School of Medicine and Faculty of Medicine Research Ethics Committee of The University of Tokyo approved the study protocol (No. 2023058NI‐(1)).

### Participants

Participants were recruited from registered panel members of an online survey company. The registered panel included over 5.6 million participants, encompassing a diverse array of sociodemographic backgrounds to ensure national representation. The panelists had the option of not responding to any part of the questionnaire and the option of discontinuing the survey at any point. The inclusion criteria for participants were as follows: (1) Japanese females, (2) aged 20–39 years, (3) with no prior history of pregnancy, (4) employed full‐time, (5) had not used contraception during the previous year, and (6) did not have PMDD during the baseline survey. The exclusion criterion was the presence of invalid or inconsistent responses. Potential participants were invited via email based on their registered sex and age information. They were provided with online information explaining the purpose and procedures of the study. Informed consent was obtained by asking participants to read the explanation and indicate their agreement by checking a consent box on the online form and submitting it. No personally identifiable information was collected. The survey was closed once the target number of respondents was reached. The respondents were invited to participate in the follow‐up survey 6 months later.

### Measures

Emergence of PMDD symptoms was assessed using the self‐report PMDD scale,^19^ which was designed based on the diagnostic criteria of the DSM‐IV^20^ and its Japanese translation.^21^ This scale consists of two components: Section A comprises 12 items assessing the intensity of the respondent's PMDD symptoms (e.g., feeling depressed or hopeless; “I feel like I can't control myself”), with responses ranging from 1 (“none”) to 4 (“severe”). Participants who reported mild, moderate, or severe symptoms in their answers to any questions in Section A advanced to Section B, which comprises five items evaluating the effects of symptoms on school or occupational performance, housework, and interpersonal relationships. Respondents who reported at least one mood symptom as being “severe,” classified their symptoms as “moderate” or “severe” for at least four items in Section A, and “severe” for at least one item in Section B, were considered to have PMDD and were selected. The Japanese version of the scale was validated elsewhere.^19^

As exposure variables, job demands, job control, coworker support, supervisor support, and workplace rewards (monetary/status reward, esteem reward, and job security) were assessed using the New Brief Job Stress Questionnaire (New BJSQ), which was tested for its reliability and validity.^22^, ^23^ The job demands and job control subscales consisted of three items, rated on a 4‐point Likert scale ranging from 1 (“very much so”) to 4 (“not at all”). The workplace reward subscales—monetary/status reward, esteem reward, and job security—each consisted of a single item, also rated on the same 4‐point scale. Supervisor support and coworker support were each measured by three items rated on a 4‐point scale, from 4 (“extremely”) to 1 (“not at all”). A higher score indicates a higher degree.

Psychological distress was measured by the Kessler 6 psychological distress scale (K6).^24^, ^25^ This scale has six items with a 5‐point Likert scale. The overall score was determined by aggregating all elements (range: 0–24). High scores indicated high psychological distress. A score of more than 5 was classified as the presence of distress.^26^, ^27^ The Japanese version of the K6 scale was evaluated for its validity and reliability.^25^

Demographic variables including age, educational attainment (high school, vocational/college, undergraduate, and graduate), marital status (single, married, and bereaved/divorced), smoking experience (never, former, and current), individual income (under JPY 4 million, JPY 4–10 million, and over JPY 10 million), occupational status (full‐time regular employee/full‐time non‐regular employee), size of company (<50 employees, 50–299 employees, 300–999 employees, and over 1000 employees), history of gynopathy (yes, no), and body mass index (BMI).

### Statistical analyses

Demographic characteristics were summarized for the participants according to psychological distress levels (K6 < 5 and K6 ≥ 5). For the main analyses, we evaluated the association between job demands and job resources and the emergence of PMDD symptoms by calculating odds ratios (ORs) and 95% confidence intervals (CIs) by logistic regression analysis. The analyses were conducted through two models: a crude model (Model 1), which evaluated univariate associations, and an adjusted model, which assessed multivariate associations after adjusting for exposure and demographic variables. In addition, we conducted the same analyses stratified by psychological distress level (i.e., K6 < 5 and K6 ≥ 5). All statistical analyses were performed using IBM spss Statistics for Windows, version 30.0 (IBM Corp., Armonk, NY, USA), and statistical significance was set at *p* < 0.05.

## RESULTS

**Figure 1:**
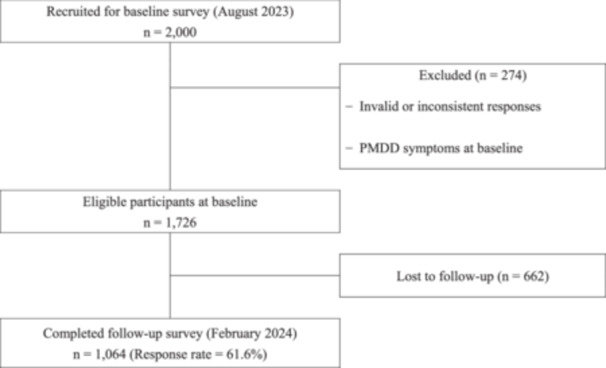
Participant flowchart.

Of the 2000 female workers who completed the baseline survey, 274 respondents with PMDD‐like symptoms exceeding the threshold on the PMDD scale were observed, and no invalid responses were excluded. Figure 1 shows the participants' flowchart. A total of 1064 participants who completed the follow‐up survey were included in this study (response rate = 61.6%). Table 1 represents the characteristics of the participants overall, with K6 < 5 (*n* = 519) and K6 ≥ 5 (*n* = 545). The average age of the participants was 35 years (SD = 6.4) in the K6 < 5 group and 35.4 (SD = 6.4) in the K6 ≥ 5 group, respectively. Approximately 82.8% (*n* = 881) were single, and 82.0% (*n* = 872) had no history of gynopathy. At the 6‐month follow‐up survey, 20 (3.7%) of the participants with K6 ≥ 5 and 5 (1.0%) of those with K6 < 5 showed emergence of PMDD symptoms.

**Table 1:** Demographic characteristics (*n* = 1064).

	Overall (*n* = 1064)		K6 < 5 (*n* = 519)		K6 ≥ 5 (*n* = 545)		
	*N* (%)	Mean (SD)	*N* (%)	Mean (SD)	*N* (%)	Mean (SD)	*p* *
Age		34.9 (6.2)		35.4 (6.2)		34.4 (6.1)	0.010
Education							0.238
High school	140 (13.2)		73 (14.1)		67 (12.3)		
Junior college/vocational school/technical college	279 (26.2)		122 (23.5)		157 (28.8)		
Undergraduate	602 (56.6)		301 (58.0)		301 (55.2)		
Graduate	43 (4.0)		23 (4.4)		20 (3.7)		
Marital status							0.278
Unmarried	855 (80.4)		407 (78.4)		448 (82.2)		
Married	183 (17.2)		99 (19.1)		84 (15.4)		
Bereaved or divorced	26 (2.4)		13 (2.5)		13 (2.4)		
Smoking							0.044
Never	916 (86.1)		436 (84.0)		480 (80.7)		
Previously	68 (6.4)		43 (8.3)		105 (19.3)		
Current	80 (7.5)		40 (7.7)		40 (7.3)		
Income							0.281
<JPY 4 million	657 (61.7)		309 (59.5)		348 (63.9)		
JPY 4–10 million	392 (36.8)		201 (38.7)		191 (35.0)		
Over JPY 10 million	15 (1.4)		9 (1.7)		6 (1.1)		
Occupational status							0.961
Full‐time regular employee	919 (86.4)		448 (86.3)		471 (82.2)		
Full‐time non‐regular employee	145 (13.6)		71 (13.7)		84 (15.4)		
Size of company							0.217
<50	255 (24.0)		134 (25.8)		121 (22.2)		
50–299	262 (24.6)		132 (25.4)		130 (23.9)		
300–999	189 (17.8)		81 (15.6)		108 (19.8)		
Over 1000	358 (33.6)		172 (33.1)		186 (34.1)		
History of gynopathy							0.289
Yes	192 (18.0)		87 (16.8)		105 (19.3)		
No	872 (82.0)		432 (83.2)		440 (80.7)		
BMI		20.5 (3.8)		20.5 (4.1)		20.6 (3.5)	0.787
Emergence of PMDD symptoms at T2							0.004
Yes	25 (2.3)		5 (1.0)		20 (3.7)		
No	1039 (97.7)		514 (99.0)		525 (96.3)		

Abbreviations: BMI, body mass index; K6, Kessler 6 psychological distress scale; PMDD, premenstrual dysphoric disorder.

* *p* for difference between K6 < 5 and K5 ≥ 5.

Tables 2‐1, 2‐2, 2‐3, and 2‐3 show the results of both the crude and adjusted models for the association between job demands and job resources at baseline and the emergence of PMDD symptoms at 6‐month follow‐up among Japanese female workers with overall participants, with K6 < 5, and with K6 ≥ 5, respectively. In the results of Model 2, job control was positively associated with the emergence of PMDD symptoms among overall participants (OR = 1.26, 95% CI [1.03, 1.54]) and participants with K6 ≥ 5 (OR = 1.37, 95% CI [1.07, 1.75]). For the participants with K6 ≥ 5, job stability was negatively associated with the emergence of PMDD symptoms (OR = 0.48, 95% CI [0.27, 0.84]).

**Table 2‐1:** Association between job workgroup resource and organizational resource and emergence of premenstrual dysphoric disorder (PMDD) symptoms among Japanese female workers (*n* = 1064).

	Model 1		Model 2	
	OR	*p*	OR	*p*
Job demand	1.39	0.129	1.40	0.174
Job control	1.14	0.154	1.26	0.025*
Supervisor support	0.84	0.081	0.93	0.605
Colleague support	0.85	0.079	0.94	0.609
Economical reward	0.57	0.019	0.78	0.418
Respect reward	0.52	0.007*	0.68	0.289
Stableness	0.63	0.030*	0.70	0.100

*Note*: Model 1: crude model. Model 2: adjusted by age, marital status, educational status, size of company, occupation, body mass index (BMI), smoking, and history of gynopathy.

Abbreviation: OR, odds ratio.

* *p* < 0.05.

**Table 2‐2:** Association between job workgroup resource and organizational resource and emergence of premenstrual dysphoric disorder (PMDD) symptoms among Japanese female workers of K6 < 5 (*n* = 519).

	Model 1		Model 2	
	OR	*p*	OR	*p*
Job demand	1.54	0.372	1.36	0.596
Job control	0.92	0.654	0.99	0.967
Supervisor support	0.91	0.656	1.18	0.614
Colleague support	0.82	0.327	0.74	0.311
Economical reward	0.67	0.441	1.21	0.797
Respect reward	0.61	0.345	0.81	0.828
Stableness	2.09	0.312	2.69	0.239

*Note*: Model 1: crude model. Model 2: adjusted by age, marital status, educational attainment, size of company, occupation, body mass index (BMI), smoking, and history of gynopathy.

Abbreviation: OR, odds ratio.

**Table 2‐3:** Association between job workgroup resource and organizational resource and emergence of premenstrual dysphoric disorder (PMDD) symptoms among Japanese female workers of K6 ≥ 5 (*n* = 545).

	Model 1		Model 2	
	OR	*p*	OR	*p*
Job demand	1.10	0.707	1.13	0.688
Job control	1.28	0.023*	1.37	0.011*
Supervisor support	0.88	0.266	0.92	0.626
Colleague support	0.93	0.500	1.07	0.659
Economical reward	0.61	0.072	0.72	0.368
Respect reward	0.58	0.050	0.65	0.333
Stableness	0.59	0.034*	0.48	0.010*

*Note*: Model 1: crude model. Model 2: adjusted by age, marital status, educational status, size of company, occupation, body mass index (BMI), smoking, and history of gynopathy.

Abbreviation: OR, odds ratio.

* *p* < 0.05.

## DISCUSSION

This study aimed to examine the longitudinal association between psychosocial factors in the workplace at baseline and the emergence of PMDD symptoms at 6‐month follow‐up among female workers in Japan, with particular attention to the status of psychological distress. As hypothesized, higher job stability prevented the emergence of PMDD symptoms among participants with high psychological distress (K6 ≥ 5). In contrast, contrary to our hypothesis, higher job control was significantly related to the emergence of PMDD symptoms among overall participants, and particularly those with high psychological distress (K6 ≥ 5).

As hypothesized, job stability was negatively associated with the emergence of PMDD symptoms 6 months later among individuals with high psychological distress. This finding aligns with the ERI model, suggesting that perceived security and predictability in the workplace can serve as critical protective factors for emotionally vulnerable workers. This is also consistent with a recent observational study reporting that job security mitigates work‐related stress and ultimately promotes better mental health.^28^ The authors noted that employees in secure jobs are more likely to prioritize their well‐being and take sick or medical leave when needed, rather than feel pressured to work while unwell.^28^ In addition, a previous review provided robust evidence that job insecurity functions as a significant stressor linked to various negative outcomes, including poor psychological well‐being.^29^ In this context, job stability may help serve as a buffer against the uncertainty and stress that exacerbate mood‐related symptoms in individuals with PMDD symptoms, particularly among workers with elevated psychological distress.

Contrary to the hypothesis, this study showed a positive association between job control and the emergence of PMDD symptoms at 6 months of follow‐up. This result was inconsistent with previous studies.^8^, ^11^, ^13^ While job control is generally considered a beneficial psychological resource, it may not function as a stress buffer for highly distressed workers; instead, it may become an additional kind of pressure when the cognitive and emotional demands associated with autonomous decision‐making exceed available coping resources.^30^ From a theoretical perspective, this result does not necessarily contradict the DCS model but instead highlights its conditional nature. The DCS model assumes that decision latitude buffers stress primarily when accompanied by adequate social support.^16^ In the absence of such conditions, job control may be experienced less as perceived control and more as responsibility overload. Consistent with this interpretation, a longitudinal study suggested that autonomy, despite its prevailing strain‐reducing effects, could be associated with demands that may impair well‐being.^31^ Recent research suggests that autonomy can increase strain when there are insufficient organizational resources, supervisory guidance, or problem‐solving ability.^32^ This distinction between perceived control and control accompanied by heightened responsibility may help explain why job control functioned as a risk‐enhancing rather than protective factor in the present study. Additionally, Lu et al. (2003) found that the mental health benefits of work autonomy were observed only among male employees and females in higher occupational classes, whereas females in lower occupational positions did not gain the same psychological benefits.^33^ Similarly, Abdel Hadi et al. (2025) reported that individuals with low trait self‐control experienced worsened subjective well‐being, specifically, greater anxiety and fatigue, when exposed to high job control.^34^ Job control may act as a source of stress for female workers who experience menstrual‐related symptoms that impair self‐regulation, as well as for those in positions of lower occupational status.

Among participants with lower levels of psychological distress (K6 < 5), no significant associations were observed between psychosocial factors in the workplace and the emergence of PMDD symptoms. Consistent with the previous study,^15^ this may indicate that the impact of psychosocial factors in the workplace on the emergence of PMDD symptoms is modulated by baseline mental health status. For individuals with lower psychological distress, workplace conditions may play a less influential role in symptom expression.

This study has several limitations. First, although this study employed a 6‐month follow‐up period, the diagnostic criteria for PMDD outlined in the DSM‐5‐TR require that symptoms related to the menstrual cycle be present in most cycles over the past year. Therefore, the shorter observation window in this study does not fully align with these criteria. Accordingly, the outcome in this study should be interpreted as PMDD symptoms rather than a formal diagnostic outcome. Second, we were unable to exclude other psychiatric conditions with overlapping symptoms. According to the DSM‐5‐TR, PMDD should not be diagnosed if another disorder better explains symptoms. As comorbidities were not assessed in this study, some participants may have had underlying psychiatric conditions. Future studies should include more comprehensive diagnostic screening. Third, the emergence of PMDD symptoms was assessed via self‐report measures, which may be subject to reporting bias. Fourth, although the longitudinal design allows for temporal analysis, causality cannot be definitively established. In addition, because all variables were assessed at only two time points, reverse causality cannot be ruled out. For example, subclinical premenstrual mood symptoms may have influenced participants' perceptions of psychosocial factors in the workplace, such as job control. Fifth, multiple psychosocial factors were examined without formal correction for multiple testing. Given the relatively small number of participants with PMDD symptoms (*n* = 25), the possibility of Type I error cannot be excluded, and the findings should be interpreted as exploratory. Sixth, this study was an online survey, and the sample consisted of full‐time Japanese female workers without a history of childbirth or hormonal medication use, which may limit generalizability to other populations. Finally, potential confounding factors such as coping style, social support outside the workplace, or hormonal variations were not controlled.

## PRACTICAL IMPLICATIONS

These findings have important implications for mental health strategies in the workplace. Ensuring stable employment conditions may serve as a buffer against the escalation of PMDD symptoms among female workers experiencing elevated psychological distress. Providing job security may not only reduce stress but also encourage employees to seek help or take leave without fear of negative consequences, thereby promoting early intervention and better symptom management. While increasing job control is often promoted as beneficial, our results suggest that a high level of job control may not be universally protective, especially among female workers experiencing psychological distress. Interventions aimed at reducing PMDD symptoms should consider tailoring work demands and responsibilities based on individual stress levels. Organizations should also provide ongoing mental health support to help female workers manage stress across hormonal cycles.

## CONCLUSION

This study investigated the longitudinal associations between psychosocial factors in the workplace and the emergence of PMDD symptoms among female workers in Japan, with a focus on the moderating role of psychological distress. The findings suggest that higher job stability is negatively associated with the emergence of PMDD symptoms among female workers with high psychological distress, while job control is positively associated in the overall sample and among workers with high psychological distress. No significant associations were observed among workers with lower psychological distress. Ensuring job stability might be a measure to prevent PMDD symptoms, especially for female workers with high psychological distress.

## AUTHOR CONTRIBUTIONS

Natsu Sasaki conceived and designed the study, supervised the research process, and contributed to data collection. Mako Iida, Kazuhiro Watanabe, and Natsu Sasaki organized the study design. Mako Iida conducted the data analysis and drafted the manuscript. Natsu Sasaki, Kazuhiro Watanabe, Miho Egawa, Yuka Ito, Yoshiaki Kanamori, Rikako Tsuji, and Daisuke Nishi critically revised the manuscript. All authors approved the final version of the manuscript for publication.

## CONFLICT OF INTEREST STATEMENT

The first author (M.I.) is employed by Fujitsu Japan Limited, which has no relation to the content or findings of this research; the company did not fund, support, or influence this study in any way. The other authors (K.W., M.E., Y.I., Y.K., R.T., D.N., and N.S.) declare that there is no conflict of interest regarding the publication of this article.

## ETHICS APPROVAL STATEMENT

The Graduate School of Medicine and Faculty of Medicine Research Ethics Committee of The University of Tokyo approved the study protocol (No. 2023058NI‐(1)).

## PATIENT CONSENT STATEMENT

Informed consent was obtained by asking participants to read the explanation and indicate their agreement by checking a consent box on the online form and submitting it.

## CLINICAL TRIAL REGISTRATION

Not applicable.

## Data Availability

The data that support the findings of this study are available on request from the corresponding author. The data are not publicly available due to privacy or ethical restrictions.
